# Evaluation of Renal Function after Partial Nephrectomy and Detection of Clinically Significant Acute Kidney Injury

**DOI:** 10.3390/medicina58050667

**Published:** 2022-05-17

**Authors:** Jurijus Makevičius, Albertas Čekauskas, Arūnas Želvys, Albertas Ulys, Feliksas Jankevičius, Marius Miglinas

**Affiliations:** 1 Institute of Clinical Medicine, Faculty of Medicine, Clinic of Gastroenterology, Nephrourology and Surgery, Vilnius University, M. K. Čiurlionio Str. 21/27, LT-03101 Vilnius, Lithuania; albertas.cekauskas@santa.lt (A.Č.); arunas.zelvys@santa.lt (A.Ž.); feliksas.jankevicius@santa.lt (F.J.); marius.miglinas@santa.lt (M.M.); 2 Center of Urology, Vilnius University Hospital Santaros Klinikos, Santariškių Str. 2, LT-08660 Vilnius, Lithuania; 3 Departament of Oncourology, National Cancer Institute, Santariškių Str. 1, LT-08661 Vilnius, Lithuania; albertas.ulys@nvi.lt; 4 Center of Nephrology, Vilnius University Hospital Santaros Klinikos, Santariškių Str. 2, LT-08661 Vilnius, Lithuania

**Keywords:** acute kidney injury, partial nephrectomy, intraoperative hypotension, neutrophil to lymphocyte ratio, clinically significant postoperative AKI, kidney dysfunction

## Abstract

*Background and Objectives*: Consequences of partial nephrectomy (PN), intraoperative hypotension (IOH) and postoperative neutrophil to lymphocyte ratio (NLR) may cause postoperative acute kidney injury (AKI) and in long-term-chronic kidney disease (CKD). Our study aimed to identify the AKI incidence after PN, to find clinically significant postoperative AKI and renal dysfunction, and to determine the predictor factors. *Materials and Methods*: A prospective observational study consisted of 91 patients who received PN with warm ischemia, and estimated preoperative glomerular filtration rate (eGFR) ≥ 60 mL/min and without abnormal albuminuria. *Results*: 38 (41.8%) patients experienced postoperative AKI. Twenty-one (24.1%) patients had CKD upstage after 1 year follow-up. Sixty-seven percent of CKD upstage patients had AKI 48 h after surgery and 11% after 2 months. All 15 (16.5%) patients with CKD had postoperative AKI. With IOH, OR 1.07, 95% CI 1.03–1.10 and *p* < 0.001, postoperative NLR after 48 h (OR 1.50, 95% CI 1.19–1.88, *p* < 0.001) was the major risk factor of AKI. In multivariate logistic regression analysis, the kidney’s resected part volume (OR 1.08, 95% CI 1.03–1.14, *p* < 0.001) and IOH (OR 1.10, 95% CI 1.04–1.15, *p* < 0.001) were retained as statistically significant prognostic factors for detecting postoperative renal dysfunction. The independent risk factor for clinically significant postoperative AKI was only IOH (OR, 1.06; *p* < 0.001). Only AKI with the CKD upstage group has a statistically significant effect (*p* < 0.0001) on eGFR 6 and 12 months after surgery. *Conclusions*: The presence of AKI after PN is not rare. IOH and NLR are associated with postoperative AKI. The most important predictive factor of postoperative AKI is an NLR of over 3.5. IOH is an independent risk factor for clinically significant postoperative AKI and together with kidney resected part volume effects postoperative renal dysfunction. Only clinically significant postoperative AKI influences the reduction of postoperative eGFR after 6 and 12 months.

## 1. Introduction

Kidney cancer is a neoplasm of renal parenchyma and the most common histopathological subtype of the kidney is renal clear cell carcinoma [1]. According to GLOBOCAN estimates, 431,288 new kidney cancer cases were diagnosed in 2020 which led to 179,368 deaths [2]. The highest incidence rates of kidney cancer in Europe were observed in Czech Republic, Lithuania and Slovakia, and the highest mortality rates from kidney cancer were observed in Lithuania, Czech Republic and Latvia [3]. Extensive use of visual diagnostic technologies, such as ultrasound, computed tomography (CT) scans, magnetic resonance imaging (MRI) and others led to more diagnoses of small localized kidney masses [4,5]. According to the National Lithuanian Cancer register data, the highest increase in incidence was observed for stage I kidney cancers [6]. Until 2010, the majority of urologists performed radical nephrectomy (RN) without considering the size of the tumor because absolute (anatomical or functional solitary kidney) indications of partial nephrectomy (PN), compared with elective indications (localized unilateral tumor with a healthy contralateral kidney), appeared to show an increase in complication rates and a risk of the development of locally recurrent disease [7]. Head-to-head comparison of RN with PN showed the same result with reference to 5-year recurrence and cancer free survival [8,9,10]. It was also observed that in the PN group, renal function (RF) was better preserved [11,12]. Today, PN is the golden nephron-sparing surgery standard for localized kidney masses [13]. In most cases during PN surgery, the renal artery is normally clamped to block blood flow. Such a surgical approach allows us to perform kidney resection and close parenchymal defects in a bloodless and better visible field when the tumor is large, close to the kidney collecting system, with a complex structure and deep parenchymal invasion [14]. The prolonged warm ischemia followed by reperfusion causes necrosis destroying the proximal tubules of the outer stripe of the medulla and impacts renal function [15]. However, there are more centers where PN warm ischemia time is reduced considering surgery experience, but there are still acute kidney injury (AKI) cases [16,17].

Intraoperative hypotension (IOH), caused by low systemic vascular resistance during anesthesia, parenchymal injury to the renal system and blood loss are all common events during PN and may lead to a sudden and temporary loss of RF. Recently published data showed that, besides creatinine, neutrophil to lymphocyte ratio (NLR) can be a practical predictor of AKI and associated with the reduced survival rate of patients who underwent PN [18,19,20]. Additionally, NLR can be used for early AKI detection as a reflecting inflammation condition after surgical kidney injury. Published data also showed the association between AKI and IOH during cardiac and non-cardiac surgery [21,22]. However, studies also showed that it is safe to perform PN without artery clamping by using controlled IOH anesthesia to preserve RF [23,24]. Moreover, there are no data available about postoperative NLR association with AKI after PN.

The aim of our study was to identify the AKI incidence after PN due to renal lesion, and find clinically significant postoperative AKI and renal dysfunction. Determine the predictor factors of those conditions among patients with estimated glomerular filtration rate (eGFR) ≥ 60 mL/min, no presence of abnormal albuminuria and renal failure history.

## 2. Materials and Methods

A prospective observational study was conducted in Vilnius University Hospital Santaros Klinikos between January 2018 and December 2019. The study was approved by the Vilnius Regional Biomedical Research Ethics Committee (approval number 158200-16-882-389) 13 December 2016 and the State Data Protection Inspectorate. The study was conducted in accordance with the Declaration of Helsinki.

### 2.1. Study Inclusion Criteria

The study population consisted of individuals over 18 years old, on whom were performed open or laparoscopic PN with warm ischemia due to kidney masses with preoperative eGFR by the Chronic Kidney Disease Epidemiology Collaboration (CKD-EPI) equal to or more than 60 mL/min/1.73 m^2^. eGFR = 141 × min(S_cr_/κ, 1)^α^ × max(S_cr_/κ, 1)^−1.209^ × 0.993^Age^ × 1.018 [if female] × 1.159 [if African American], where: S_cr_ is serum creatinine in µmol/L, κ is 61.9 for females and 79.6 for males, α is −0.329 for females and −0.411 for males, min indicates the minimum of S_cr_/κ or 1, and max indicates the maximum of S_cr_/κ or 1.

### 2.2. Study Exclusion Criteria

We excluded patients with abnormal albuminuria, history of AKI, CKD and kidney surgery, pregnant or planning pregnancy because it would be difficult to clearly understand the nature of renal function decline. Patients with hyperkalemia without uremia and hyperuricemia were also excluded because of the initial loss of nephron function due to kidney disease resulting in renal retention of potassium and uric acid. Moreover, we excluded only patients with unregulated hypertension and diabetes mellitus avoiding faster nephropathy.

### 2.3. Anesthetic Management

Endotracheal intubation was used. Fentanyl infusion was performed before induction (0.5–1 mcg/kg bolus in 30–60 s) and it continued (0.015–1 mcg/kg/lBW/min) during PN. Induction continued with propofol 1–2 mg/kg. For relaxation, 1 mg/kg/lBW rocuronium was used. Sevoflurane 1 minimum alveolar concentration, O_2_/air fraction 0.5, with FiO_2_ 55%, and inspiratory fresh gas flow of 1 L/min were used. In the postoperative period, 1 mg/kg IV tramadol and 75 mg IM diclofenac were used as painkillers. During open PN extra spinal anesthesia 7.5 mg of 0.5% heavy bupivacaine was administered in combination with the 0.1–0.2 mg morphine sulfate dose after local anesthesia with lidocaine 40 mg. Infusion by sodium chloride 0.9% 2000–3500 mL was performed during all surgery time.

### 2.4. Surgical Procedure

All patients’ treatment options were chosen under the same protocol. PN was offered to patients with tumor ≤ 7 cm (T1 stage).

Open partial nephrectomy:

The position was a full-flank position and an extended lumbar incision was made above the 11th or 12th rib. The main artery was clamped using a bulldog clamp. Incision of the renal capsule was made sharply by a scalpel. The tumor was separated bluntly from the surrounding parenchyma. The absorbable gelatin sponge for hemostasis was placed on the cut surface and Vicryl sutures were used to approximate the remaining renal parenchyma with a mattress suture.

Laparoscopic partial nephrectomy:

Performed by a transabdominal approach, four ports were placed when the lesion was on the left side and an additional one if it was on the right. The main renal artery was clamped with a bulldog clamp. For resection of the tumor, scissors were used in warm ischemia time. Hemostasis was achieved by central suturing with non-absorbable clips and hemostatic sponge.

### 2.5. Data and Definitions

We collected clinical and laboratory data, including age, gender, body mass index (BMI), white blood cells (WBC), neutrophils and lymphocytes percent, calculated neutrophil to lymphocyte ratio (NLR) and divided into two groups (more than 3.5 (increased) and more than nine (severely increased)), serum creatinine (sCr), urine albumin-creatinine ratio (uACR), fasting glucose, total cholesterol, low-density lipoprotein cholesterol, high-density lipoprotein cholesterol, triglycerides and spot urine, standardized nephrometry scoring system (R.E.N.A.L. Nephrometry Score) to quantify the anatomical characteristics of renal masses on CT imaging before PN. We also analyzed surgery data such as duration, ischemia and IOH time. The resected kidney parts were fixed with formalin and grossed by a surgical pathologist according to the departmental standard operating procedure. The gross pathologic measurements of the resected part and kidney tumors were performed using a standard plastic centimeter scaled ruler with 0.1-cm markings. Measurements were recorded in three dimensions after formalin inflation and fixation and serial sectioning of the surgical specimen.

RF was grouped using CKD class (G1 ≥ 90 mL/min, G2, 60–89 mL/min; G3, 30–59 mL/min; G4, 15–29 mL/min; G5, <15 mL/min) preoperatively and 12 months after surgery. CKD upstage was defined as a new-onset developed lowest CKD class 12 months from PN. CKD was defined as eGFR < 60 mL/min/1.73 m^2^ for more than 3 months and abnormal albuminuria as albumin creatinine ratio in a single urine sample of ≥3 mg/mmol.We defined AKI as a blood creatinine increase of 1.5 times within 48 h: I stage -sCr 1.5–1.9 times higher baseline; II stage-sCr 2.0–2.9 times higher baseline; III stage-sCr 3.0 times higher baseline. Metabolic syndrome (MetS) was presented if three or more of the following five criteria were met: waist circumference ≥ 102 cm in men or ≥88 cm in women, blood pressure ≥ 130/85 mmHg, fasting triglyceride (TG) level ≥ 1.7 mmol/L, fasting high-density lipoprotein (HDL) cholesterol level less than 1.03 mmol/L in men or 1.29 mmol/L in women and fasting blood glucose ≥ 5.6 mmol/L.

**Figure 1:**
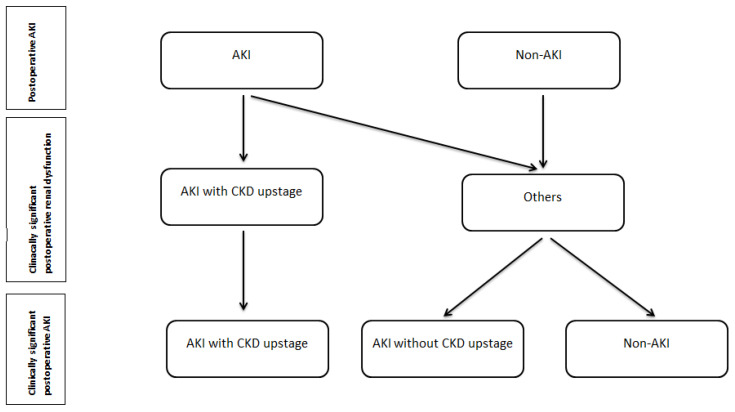
The study groups formation. Abbreviations: AKI–acute kidney injury; CKD–chronic kidney disease.

Patients were divided into three groups according to CKD upstage status and AKI, see Figure 1. The first group, clinically significant postoperative AKI, we defined as the presence of AKI in CKD upstage group patients. Another two groups, non-clinically significant postoperative kidney dysfunction, were defined as non CKD upstage patients with/without postoperative AKI. The second group was AKI patients without CKD upstage and the third was the non-AKI group.

### 2.6. Statistics

Statistical analysis was performed using the R statistical software package V 4.0.2 (2020-06-22) (© The R Foundation for Statistical Computing), Rstudio Version 1.3.959 © 2009–2020 RStudio, PBC, IBM SPSS Statistics V.23, G*Power V. 3.1.9.4 Universität Düsseldorf, Germany. Interval and ratio variables were described by minimum (Min) and maximum (Max) values, means and standard deviations (SD), medians, first quartiles (Q1) and third quartiles (Q3), and interquartile deviations (IQR 75%). Shapiro–Wilk and Kolmogorov–Smirnov (K–S) tests were used to check the data for normality. Nominal and ordinal variables were characterized by frequencies and percentages across the corresponding subset of the sample. To assess the statistically significant difference among the independent groups we used Fisher’s chi-squared test.

Univariate analyses were performed with postoperative AKI and clinically significant renal dysfunction as the dependent variables and sociodemographic factors (age, gender, BMI, CCI, MetS), laboratory data (preoperative eGFR and uACR, postoperative NLR), surgery data (ischemia and IOH times, blood loss, resected part, tumor and removed parenchymal volumes) as the independent variables, but only the statistically significant (*p* < 0.05) were shown.

In order to assess a statistically significant influence of relevant independent variables (age, gender, BMI, CCI, MetS, preoperative eGFR, postoperative NLR on the postoperative AKI, ischemia and IOH times, blood loss, resected part, tumor and removed parenchymal volumes) on postoperative AKI and clinically significant postoperative renal dysfunction, we created models based on logistic regression equations with optimization to evaluate the independent effects of each covariate by controlling the effects of other variables. The receiver operating characteristic (ROC) curve was used to determine the optimal cut-off value. The same variables with *p* < 0.05 in the univariate analysis were incorporated into the multivariate analysis for predicting significant postoperative AKI.

To measure the effect size between eGFR after 6 and 12 months and postoperative AKI clinically significant postoperative renal dysfunction groups, we used the Hedge effect size. We will assume that when r_S_ = 0.1 − <0.3 we have a small effect, when r_S_ = 0.3 − <0.5, we have a moderate effect and, when r_S_ ≥ 0.5, we have a large effect. To measure the effect size between eGFR after 6 and 12 months of clinically significant postoperative AKI (AKI with CKD), AKI without CKD and non-AKI groups, we used the Omega squared (ω2) effect size. We will assume that, when ω2 = 0.1 − <0.06 we have a small effect, when ω2 = 0.06 − <0.14, we have a moderate effect and, when ω2 ≥ 0.14, we have a large effect. To increase the accuracy of estimates, in addition, we used Bayesian parameter estimation and Bayesian hypothesis testing.

Relationships between variables were considered statistically significant when the *p*-value was less than 0.05 (*p* < 0.05) and a statistical test power of 1-ß was equal to 0.95 (1-ß = 0.95).

### 2.7. Baseline Characteristics

A total of 91 patients were agreed on and included in the study. The demographic and clinical characteristics of study participants are summarized in Table 1. Perioperative, intraoperative and postoperative laboratory data are summarized in Supplementary Table S1: Study patients perioperative, intraoperative and postoperative laboratory data.

## 3. Results

Out of 91 study patients, 38 (41.8%) had developed postoperative AKI, 36 patients had I stage and 2-with II stage, 21 (24.1%) patients had CKD upstage 1 year from PN. Sixty percent of CKD upstage group patients had AKI 48 h after surgery. After 2 months, the AKI amount decreased from 38 cases to 11. All 15 (16.5%) patients with CKD after 12 months follow up had AKI.

The median (IQR) age of AKI patients included in the study was 69.0 (13.0) years. The median preoperative eGFR of AKI patients was lower than in non-AKI—78.5 (21.5) vs. 93.0 (11.0) mL/min 1.72 m^2^, *p* < 0.001. There were more AKI patients with MetS (31 (81.6%) vs. 27 (50.9), *p* = 0.004), estimated blood loss during PN (400.0 (217.5) vs. 300.0 (220.0) mL, *p* = 0.005), longer IOH time (30.0 (30.0) vs. 0.0 (10.0) min, *p* < 0.001). Moreover, a higher volume of resected kidney part (70.6 (71.0) vs. 41.0 (53.7) mL, *p* = 0.026), tumor (38.0 (51.6) vs. 18.9 (42.6), *p* = 0.048), removed parenchymal (24.5 (21.7) vs. 20.7 (15.9), *p* = 0.049) in AKI patients.

The preoperative eGFR categories between the postoperative kidney dysfunction group and the non-clinically significant postoperative kidney dysfunction group was not significantly different (*p* = 0.083), but the first group had older patients, median years (IQR) 71.0 (12.0) vs. 63.0 (13.0), *p* = 0.02. Moreover, the frequencies of the CCI score, patients with MetS, longer ischemia and IOH time, higher resected kidney’s part, removed tumor and parenchymal volume, and estimated blood loss volume during surgery were all significantly higher in the patients with clinically significant postoperative kidney dysfunction (*p* < 0.05). Upon creatinine, NLR was significantly higher in patients that developed AKI than in patients without AKI (median (IQR) 5.7 (6.5) vs. 3.3 (1.3), *p* < 0.001) and still significantly higher after clinically significant postoperative kidney dysfunction group formation (median (IQR) 4.5 (2.7) vs. 3.5 (2.8), *p* = 0.017).

### 3.1. Risk Factors for Postoperative AKI

The potential predictors of AKI after PN are summarized in Table 2.

**Figure 2:**
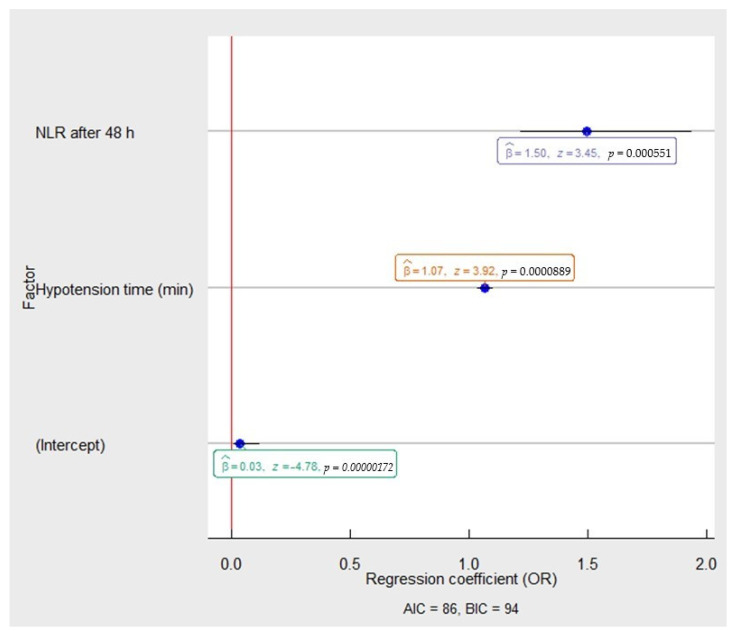
The multivariable analysis of risk factors associated with postoperative AKI in the patients after partial nephrectomy. The OR and 95% CI were measured through logistic regression. Model characteristics: X^2^ = 43.56, *p* = 0.00; Pseudo−R² (Cragg–Uhler) = 0.51; Pseudo−R² (McFadden) = 0.35. Abbreviations: NLR−neutrophil to lymphocyte ratio; OR-odds ratio: AIC−Akaike information criteria; BIC−Bayesian information criteria.

Age, estimated blood loss > 500 mL during PN, IOH and NLR > 3.5 were potential predictors for AKI development. With IOH (OR 1.07, 95% CI 1.03–1.10, *p* < 0.001), it was evident that postoperative NLR after 48 h was the major risk factor of AKI development (OR 1.50, 95% CI 1.19–1.88, *p* < 0.001), see Figure 2.

**Figure 3:**
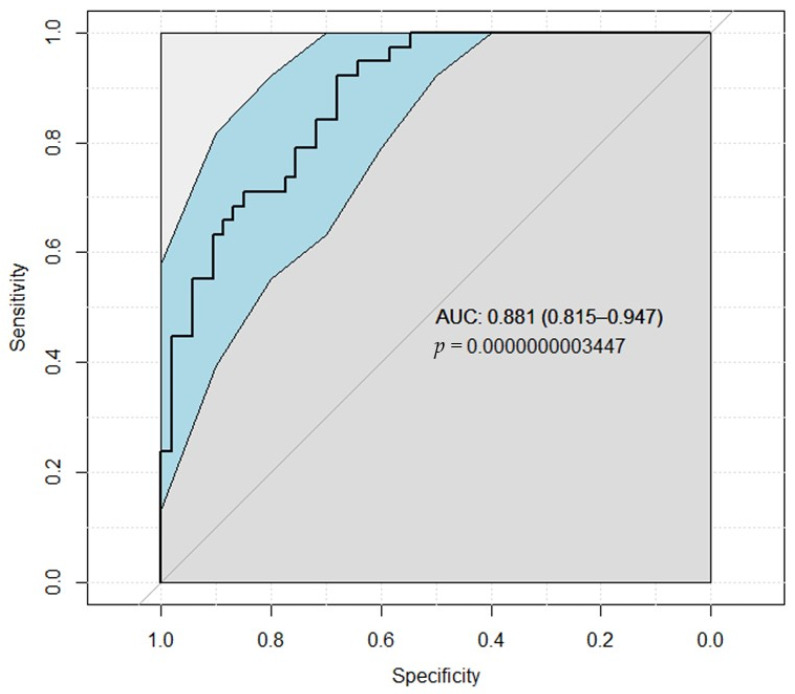
ROC analysis result for neutrophil to lymphocyte ratio and intraoperative hypotension detecting AKI. Abbreviations: AUC−area under the curve.

In the ROC analysis (Figure 3), NLR with IOH was the significant independent predictor (*p* < 0.0001) for AKI with an area under the ROC curve of 0.881 (95% CI = 0.815 to 0.947; sensitivity = 71.1%, specificity = 84.9%, positive predictive value = 77.1%, negative predictive value = 80.4%, prevalence = 41.8%).

### 3.2. Risk Factors for Postoperative Renal Dysfunction in Patients after PN

The potential predictors of clinically significant postoperative kidney dysfunction after PN are summarized in Table 3.

**Figure 4:**
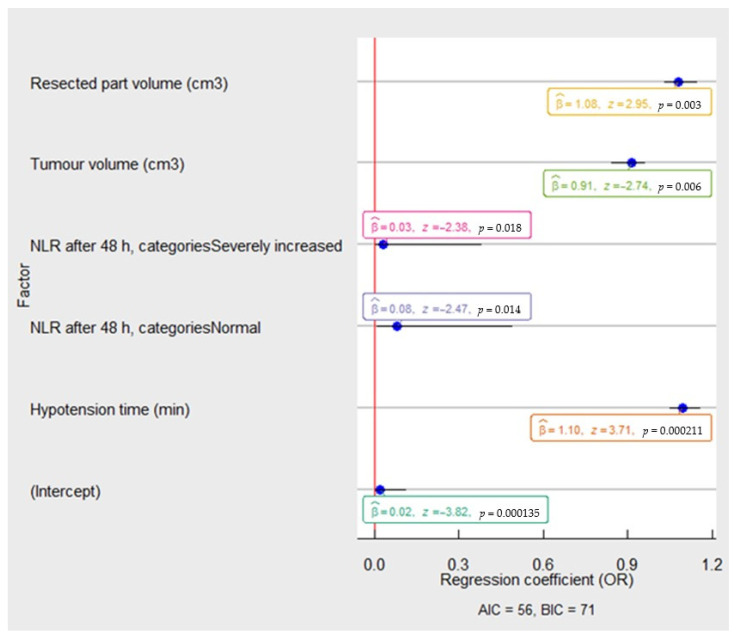
The multivariable analysis of risk factors associated with clinically significant postoperative kidney dysfunction in the patients after partial nephrectomy. The OR and 95% CI were measured through logistic regression. Model characteristics: X^2^ *=* 54.38, *p* = 0.00; Pseudo−R² (Cragg–Uhler) = 0.68; Pseudo−R² (McFadden) = 0.55. Abbreviations: NLR—neutrophil to lymphocyte ratio; OR—odds ratio: AIC—Akaike information criteria; BIC—Bayesian information criteria.

Older age, higher CCI score, estimated blood loss during PN more than 500 mL, longer intraoperative and ischemia times, higher removed parenchymal and resected part volumes were potential predictors for clinically significant postoperative kidney dysfunction development. In multivariate logistic regression analysis (Figure 4), the kidney’s resected part volume (OR 1.08, 95% CI 1.03–1.14, *p* < 0.001) and IOH (OR 1.10, 95% CI 1.04–1.15, *p* < 0.001) were retained as statistically significant prognostic factors for detecting clinically significant postoperative kidney dysfunction after PN.

**Figure 5:**
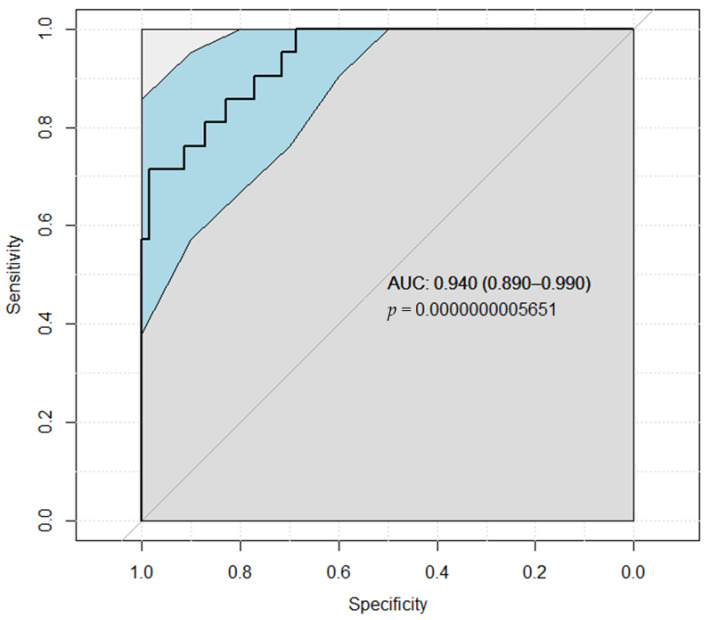
ROC analysis result for neutrophil-to-lymphocyte ratio, intraoperative hypotension, tumor and resected kidney volumes detecting postoperative kidney dysfunction after partial nephrectomy. Abbreviations: AUC−area under the curve.

IOH and higher volume of resected kidney were determined as an optimum cut-off value for predicting postoperative renal dysfunction (*p* < 0.0001), which had an area under the ROC curve of 0.940 (95% CI = 0.890 to 0.990; sensitivity = 71.4%, specificity = 95.7%, positive predictive value = 83.3%, negative predictive value = 91.8%, prevalence = 23.1%), Figure 5.

### 3.3. Risk Factors of Clinically Significant Postoperative AKI after PN

Our study found 21 patients with CKD upstage, who had AKI status after 48 h from PN. The clinical characteristics, perioperative factors, laboratory data, and outcomes of the patients with AKI and CKD upstage status are shown in Table 1. In the AKI with CKD upstage group there were statistically significant differences in the age, preoperative eGFR, preoperative uACR, MetS, ischemia time, estimated blood loss and intraoperative hypotension. Moreover, in the AKI with CKD upstage group, there were statistically significantly higher kidney‘s resected parts, tumors and removed parenchymal volumes with focal global glomerulosclerosis.

In Table 4, the multivariable analysis showed that the independent risk factor for clinically significant postoperative AKI after PN was only the intraoperative hypotension time during PN (1.06 OR, 1.06; *p* < 0.001). On the other hand, preoperative eGFR ≥ 90 mL/min and a removed kidney‘s parenchymal volume of less than 30 mL reduced the risk of clinically significant postoperative AKI.

### 3.4. The Postoperative AKI Influence on Long-Term Renal Function

**Figure 6:**
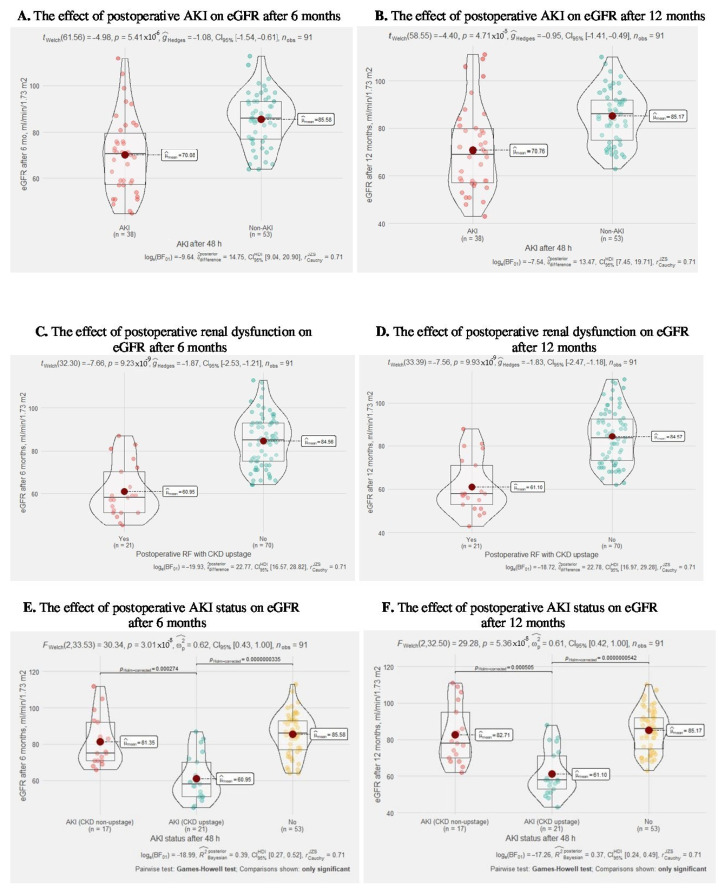
The effect of postoperative renal function on eGFR. Welch’s *t*-test revealed that, across 91 patients, although the eGFR after 6 months, mL/min/1.73 m^2^: (**A**)—were lower in AKI group after 48 h, as compared to non−AKI group. This effect was statistically significant. The effect size (g = −1.08), (*p* < 0.0001) was high, as per Cohen’s (1988) conventions. The Bayes Factor for the same analysis revealed that the data were 15 times more probable that the means are different as compared to the null hypothesis, that the means are equal; (**C**)—were lower in postoperative renal dysfunction group, as compared to non−dysfunction group. This effect was statistically significant. The effect size (g = −1.87), (*p* < 0.0001) was high, as per Cohen’s (1988) conventions. The Bayes Factor is 23; (**E**)—were lower in AKI with CKD upstage group after 48 h, as compared to non−AKI and AKI without CKD upstage groups. This effect was statistically significant. The effect size (g = −0.62), (*p* < 0.0001) was high. Welch’s *t*-test revealed that, across 91 patients, although the eGFR after 12 months, mL/min/1.73 m^2^: (**B**)—were lower in AKI group after 48 h, as compared to non−AKI group. This effect was statistically significant. The effect size (g = −0.95), (*p* < 0.0001) was high. The Bayes Factor is 13; (**D**)—were lower in postoperative renal dysfunction group, as compared to non−dysfunction group. This effect was statistically significant. The effect size (g = −1.83), (*p* < 0.0001) was high, as per Cohen’s (1988) conventions. The Bayes Factor is 23; (**F**)—were lower in AKI with CKD upstage group after 48 h, as compared to non−AKI and AKI without CKD upstage groups. This effect was statistically significant. The effect size (g = −0.62), (*p* < 0.0001) was high.

In Figure 6, we can observe that postoperative AKI cases after 48 h and postoperative kidney dysfunction after PN statistically significantly influence eGFR 6 and 12 months after surgery (both *p* < 0.0001), with a significant effect size. Moreover, splitting postoperative AKI into a clinically significant postoperative AKI group (AKI with CKD upstage) and AKI without CKD showed that only the AKI with CKD upstage group had a statistically significant effect (*p* < 0.0001) on eGFR 6 and 12 months after surgery in the compartment with the AKI group without CKD upstage and non-AKI patients. A detailed visualization of renal function is summarized in Supplementary Figure S1: The graphics of postoperative renal function on eGFR.

## 4. Discussion

Our study showed that patients with IOH are associated with clinically significant postoperative AKI after PN and NLR of over 3.5 at a 1.5 times increased risk of postoperative AKI and postoperative eGFR decline after 6 and 12 months influenced by clinically significant postoperative AKI.

RF is generally better preserved after PN than after RN due to a higher amount of nephron preservation and the preserved parenchymal volume has been detected as a primary indicator of postoperative RF [25]. However, our prospective study with patients eGFR ≥ 60 mL/min and without microalbuminuria demonstrate that some preoperative (age, eGFR) and intraoperative (hypotension, estimated blood loss) factors, associated with patient clinical characteristics (postoperative higher NLR) and technical aspects (higher resected kidney volume) of PN, showed a significant risk of the occurrence of AKI, decreased postoperative eGFR and the presence of CKD after 1 year follow-up.

In most cases of the use of conventional diagnostic criteria of AKI, the incidence of AKI after partial nephrectomy was reported to be 20–54% [26,27]. These findings are in line with our results; 42% of patients after 48 h had postoperative AKI, but only 23% had clinically significant postoperative AKI. Moreover, AKI rate depends on chosen AKI criteria definition [28,29]. Renal function decline after PN reflects incomplete recovery of the remaining kidney from surgery, ischemic insult and parenchymal volume loss together with tumor during resection. AKI after RN is closely associated with the development of CKD and increased mortality, but for PN this association has not been clearly ascertained. Previous studies reported controversial results, possibly due to difficulties with diagnosing the pure renal parenchymal injury [30,31].

Increased BMI could be an independent predictor for AKI after PN due to adherent peripheral fat which can increase the complexity of surgery by limiting kidney mobilization and isolation of the renal hilum. Sood et al.’s study from the National Surgical Quality Improvement Database, which included 141,802 patients across most common surgeries, demonstrated that overweight patients have an increased risk of complications, including kidney injury. However, in all techniques of nephrectomies BMI had no effect on perioperative complications [32]. Rosen et al. evaluated the impact of obesity on patients undergoing robotic partial nephrectomy (RPN). Results of multivariable analysis show that obesity (OR = 1.81; *p* = 0.031), male sex (OR = 1.54; *p* = 0.028), and larger tumor size (OR = 1.23; *p* < 0.001) were associated with a significant increase in the likelihood of AKI at discharge. BMI above normal weight was not associated with greater eGFR decline per month post-RPN [33]. Another study by Martini et al. showed that in the AKI group there were more patients with increased BMI, 31.3 (26.6, 36.4) vs. 29.0 (25.7, 34.0), *p* < 0.001. Based on univariate (OR = 1.04, 95% CI: 1.02, 1.06, *p* < 0.001) and multivariate analysis (OR = 1.05, 95% CI: 1.03, 1.08, *p* < 0.001), BMI emerged as a predictor of AKI [34]. The results of our study show that being overweight without CKD and albuminuria had no significant association with AKI after PN. This may suggest that PN is a safe and effective modality for patients regardless of BMI status and could influence only the duration of operation.

The MetS influence of perioperative outcomes after PN has been poorly investigated, but it can be a reason for higher AKI rates. This result is based on the presence of at least three out of five (high blood pressure, BMI ≥ 30, altered fasting glucose, low HDL-cholesterol and high triglycerides) MetS components [35]. Z. Liu et al. investigated MetS association with improved cancer-specific survival in patients with localized kidney tumors and also found that higher preoperative creatinine level was more prevalent in MetS patients; 17.6% vs. 7%, *p* < 0.001 [36]. In our study we found 58 patients with MetS. After PN this group of patients showed a mean creatinine level below the normal range. We did not demonstrate the individual ability of each MetS component due to patient limitations in this study. Only based on univariate analysis, patients without MetS had less risk of postoperative kidney dysfunction (OR = 0.22, 95% CI: 0.05, 0.73. *p* = 0.024) and postoperative AKI (OR = 0.23, 95% CI: 0.08, 0.60, *p* = 0.004). Other studies also did not indicate MetS as a direct AKI predictor, but it can strongly predict perioperative complications after PN [37]. The strength of the effect was directly proportional to the number of MetS components, even if MetS criteria had not been normally matched.

Winer et al.’s study performed an analysis of 387 patients who underwent PN and found that increases in postoperative RF occurred in a substantial number of patients. This increase was most pronounced in younger patients with higher preoperative eGFR (OR = 0.72, 95% CI: 0.65, 0.79. *p* < 0.001). Patients with lower preoperative eGFR were significantly associated with advanced age, larger tumor size, male sex, history of smoking, hypertension and longer ischemia time. Only advanced age, lower preoperative eGFR and longer ischemia time were independent predictors of decreasing renal function after surgery [38]. This study did not examine the clinical situation of patients’ characteristics such as AKI, which better describe RF and patient health. Zabor et al.’s study showed that 49% of patients after RN experienced recovery to the baseline eGFR following an initial postoperative decrease, and patients with preoperative eGFR ≥ 60 mL/min per 1.73 m^2^ were significantly more likely to recover after RF decrease [39]. In the collaborative review of the literature of renal ischemia and function after PN, the authors state that other articles strongly support the preoperative GFR and quantity of nephrons preserved as the most important determinants of functional recovery after PN because hyperfiltration of the remaining nephrons may compensate, to a certain degree, for their decreased number. Univariate analysis of our study shows that higher preoperative eGFR significantly reduces the risk of postoperative AKI (OR = 0.94, 95%, CI: 0.91, 0.97. *p* = 0.001) and renal dysfunction (OR = 0.91, 95% CI: 0.86, 0.95. *p* < 0.001), but in conjunction with other factors preoperative eGFR ≥ 90 mL/min statistically significantly reduces the risk of only clinically significant postoperative AKI (OR = 0.28, *p* = 0.01).

During PN, the main artery is routinely clamped to minimize blood loss which allows a surgeon to work in a bloodless area; it gives the ability to radically remove the tumor, safely repair the parenchymal defect and is often usable during open, laparoscopic and robotic PN. However, arterial clamping leads to a potential loss of otherwise healthy nephrons [14]. Previous studies, such as Kallingal et al. and the collaborative review of the literature by Volpe et al. have demonstrated that renal parenchymal tissue is tolerant for warm ischemia time (WIT) up to 20–30 min. During PN, prolonged ischemia time is associated with RF decline [40,41]. Porpiglia et al. used ROC curve analysis to show that WIT longer than 25 min has a chance of having a significant loss; RF is 6.5 times higher than when WIT is less than 25 min [42]. Surprisingly, decreased RF occurred within 3 months and did not change significantly after one year. Our study data did not demonstrate any significant AKI correlation with WIT, because IQR interrupting the flow of blood time in the AKI group was 15 (8.0) min. The classical strategy that limits ischemic kidney injury is the use of the selective clamping of the pertinent segmental artery only. Early unclamping of the main renal artery or hypothermia technique, also called cold ischemia (CI), during which kidney surface is cooled with ice slush, decreases renal energy expenditure due to low metabolism and partly ameliorates the adverse impact of warm ischemia and reperfusion injury [14,43]. Nevertheless, Eggener et al.’s sensitivity analysis study using absolute eGFR changes did not predict any significant differences between the CI and WIT groups [44].

All PN is performed under general anesthesia. Some surgeons used controlled IOH anesthesia during PN to preserve RF [23,24]. However, in the state of anesthesia, potential episodes of hypotension may cause kidney ischemia. It can alter renal perfusion, induce cycles of ischemia and reperfusion, oxygen supply–demand mismatch, increase oxidative damage, and increase renal and systemic inflammation—all mechanisms implicated in the development of AKI [45,46]. Moreover, IOH can activate the sympathetic nervous system, consequently releasing catecholamines and inducting the renin–angiotensin–aldosterone cascade and impairing renal oxygenation during surgery [47]. Bijker et al.’s article described 140 definitions of IOH with its frequency varying from 5% to 99% [48]. Our definition of IOH was based on a blood pressure reading lower than 90 mm Hg for the systolic and 60 mm Hg for the diastolic pressure with middle artery pressure < 70 mm Hg. Based on previous studies we suggest that even short periods of hypotension below 50 to 65 of MAP mmHg are associated with kidney injury. Our study shows that in the AKI group median IOH time was higher than in the non-AKI group; 30 (IQR 30) min vs. 0 (IQR 10) min, *p* < 0.001. Based on a multivariable analysis of our study, IOH is an independent risk factor for postoperative AKI, renal dysfunction and clinically significant postoperative AKI.

The interaction between intraoperative blood loss and the relationship between postoperative AKI was evaluated by Kawamura et al. [49]. They found that the median estimated blood loss was 314 mL in the AKI group and in the univariate analysis was significantly associated with the onset of AKI (OR = 2.65, 95% CI: 1.04, 7.64. *p* = 0.041). The multivariate model of this study revealed that high tumor complexity, but not its size, was the only independent predictor for AKI after surgery. During our study we measured blood loss by collecting samples from a pump tank and weighing operating bondage. Results showed that a median blood loss of more than 500 mL during PN could significantly increase the odds of kidney injury. We suggest that this factor is not the main predictor of AKI, but should be used in conjunction with WIT, IOH and preoperative glomerular status influence oxygenated blood distribution in the parenchyma. Moreover, we measured real tumor volume in the resected sample and resected part volume; we found no association with postoperative AKI.

During PN, the surgeon inflicts kidney parenchymal injury which causes non-bacterial inflammation. Local inflammation can play an important role in the initiation and progression of AKI after PN and hematological results could be associated with AKI. Known studies have focused on biomarkers, such as NGAL and KIM-1, to detect AKI [50]. However, NLR can be cheaper and easily calculated in clinical practice from complete blood count [51]. We did not find any prospective study about NLR after PN, so we tried to analyze it. The stage of active inflammation is not well known, but total leucocyte count provides a rough but sensitive assessment of inflammatory status. Previous studies showed that NLR is a marker of poor prognosis in several disorders such as malignancies, CKD and myocardial infarction [52]. These inflammatory findings in blood specimens can be accompanied by the glomerular inflammatory process and the activation of systemic inflammatory markers. A retrospective study by Tonyali, et al. demonstrated that the cutoff value of 3.18 for NLR associated with CKD with 39% sensitivity and 81% specificity (*p* = 0.11) and with 2.8-fold increased risk to have CKD after PN and radical nephrectomy [19]. Moreover, this is the first reported study where NLR is negatively correlated with GFR and positively correlated with CKD stage (*p* = 0.028). Our study demonstrated that an NLR higher than 3.5 predicts postoperative AKI in patients after PN (OR = 1.50, 95% CI: 1.19, 1.88. *p* < 0.001), but not clinically significant postoperative AKI. Another fact of our study is that the probability of postoperative renal dysfunction is minimal when NLR less than three or more than nine.

Twelve months of follow-up with our patients showed that 14 (15%) patients developed CKD 6 months after surgery and this amount increased to 15 cases 12 months after surgery. Comparing patients with AKI 48 h after PN with eGFR 6 and 12 months after surgery, we found that the postoperative AKI after PN is associated with lower eGFR due to the influence of clinically significant postoperative AKI.

Our study has several limitations. Firstly, the small size of the study impacts the precision of estimates and parameters of interest and makes it difficult to determine which preoperative, intraoperative variable is more closely associated with postoperative AKI. Larger studies will be required to confirm our findings. Secondly, using another AKI criterion would probably increase sample size. Furthermore, we examined IOH by non-invasive monitoring of arterial pressure and all episodes of a patient’s IOH were summarized in one attempt due to the difficult accounting of intermittent data. Lastly, our study results show renal function changes after warm ischemia PN with main artery clamping. We did not carry out long-term follow-up and evaluate long-term postoperative eGFR changes.

This study has some strengths. This is the first study of independent risk factors for postoperative renal dysfunction and the evaluation of clinically significant postoperative AKI in Lithuania. We showed the potential influence of NLR on predicted rates of postoperative AKI after PN.

## 5. Conclusions

Our study demonstrates that the presence of AKI after PN is not rare and it affects long-term RF in patients without CKD. IOH and NLR are associated with postoperative AKI in patients who underwent PN. The most important predictive factor of postoperative AKI is an NLR of over 3.5. IOH is independent risk factor for clinically significant postoperative AKI and, together with kidney resected part volume, affects postoperative renal dysfunction. Only clinically significant postoperative AKI influences the reduction of postoperative eGFR after 6 and 12 months.

## Figures and Tables

**Table 1:** Study patients demographic and clinical characteristics.

Variable	Value	AKI (CKD Upstage)	AKI (CKD Non-Upstage)	Non-AKI	*p*-Value (Fisher’s)
Age, y	Median (IQR)	71.0 (12.0)	67.5 (17.0)	62.0 (11.2)	0.023
Hospital stay, day	Median (IQR)	6.0 (2.0)	7.0 (3.0)	6.0 (2.0)	0.051
Gender	Female	11 (52.4)	5 (27.8)	22 (42.3)	0.294
	Male	10 (47.6)	13 (72.2)	30 (57.7)	
BMI, kg/m^2^	Median (IQR)	28.7 (2.9)	27.2 (5.8)	28.4 (5.6)	0.858
CCI, score	Median (IQR)	5.0 (2.0)	4.0 (2.0)	4.0 (2.0)	0.096
R.E.N.A.L. score, points	Median (IQR)	7.0 (1.0)	7.0 (1.8)	6.0 (1.2)	0.149
Metabolic syndrome	Yes	18 (85.7)	13 (72.2)	27 (51.9)	0.016
	No	3 (14.3)	5 (27.8)	25 (48.1)	
Partial nephrectomy	Laparoscopic	12 (57.1)	5 (27.8)	33 (63.5)	0.034
	Open	9 (42.9)	13 (72.2)	19 (36.5)	
Ischemia time, min	Median (IQR)	19.0 (6.0)	12.5 (5.0)	15.0 (8.5)	0.009
Ischemia time classification, min	<10	1 (4.8)	3 (16.7)	13 (25.0)	0.045
	10–20	12 (57.1)	14 (77.8)	31 (59.6)	
	>20	8 (38.1)	1 (5.6)	8 (15.4)	
eBlood loss, mL	Median (IQR)	490.0 (140.0)	300.0 (172.5)	300.0 (225.0)	<0.001
eBlood loss classifiaction, ml	≤500	11 (52.4)	16 (88.9)	48 (92.3)	<0.001
	>500	10 (47.6)	2 (11.1)	4 (7.7)	
Hypotension time during surgery, min	Median (IQR)	40.0 (15.0)	10.0 (28.8)	0.0 (10.0)	<0.001
Resected part volume, cm^3^	Median (IQR)	87.1 (53.3)	34.5 (50.3)	41.3 (54.4)	<0.001
Tumor volume, cm^3^	Median (IQR)	53.7 (35.7)	16.4 (47.5)	19.8 (43.4)	0.017
Parenchymal volume removed, cm^3^	Median (IQR)	34.7 (16.8)	14.3 (12.1)	20.7 (15.6)	<0.001
Parenchymal loss	>20 cm^3^	14 (66.7)	1 (5.6)	10 (19.2)	<0.001
	<20 cm^3^		10 (55.6)	17 (32.7)	
Parenchymal pathology	Focal global glomerulosclerosis	11 (52.4)	1 (5.6)	11 (21.2)	0.009
	None	10 (47.6)	17 (94.4)	41 (78.8)	

Abbreviations: AKI—acute kidney injury; CKD—chronic kidney disease; IQR—interquartile range; BMI —body mass index; CCI—Charlson comorbidity index; R.E.N.A.L.—renal masses nephrometry scoring system; eBlood loss—estimated blood loss; ASA—American society of anesthesiologists score; RCC—renal cell carcinoma; pTNM—pathological tumor-node-metastasis staging. *p*-values calculated for comparison of non-AKI, AKI without CKD upstage and AKI with CKD upstage cohorts.

**Table 2:** Univariate analysis of the associations between clinical characteristics and postoperative AKI persistence.

	Univariate				
Variables	AKI	Non-AKI	OR	95% CI	*p*-Value
Age	69.0 (13.0)	62.0 (11.0)	1.06	1.01–1.11	0.020
No metabolic syndrome	7 (18.4)	26 (49.1)	0.23	0.08–0.60	0.004
Preoperative eGFR	78.5 (21.5)	93.0 (11.0)	0.94	0.91–0.97	0.001
Estimated blood loss > 500 mL	12 (31.6)	4 (7.5)	5.65	1.77–21.86	0.006
Intraoperative hypotension time	30.0 (30.0)	0.0 (10.0)	1.07	1.04–1.10	<0.001
Neutrophil lymphocyte ratio > 3.5	34 (89.5)	19 (35.8)	1.57	1.12–1.92	0.001

Abbreviations: OR—odds ratio; CI—confidence interval; eGFR—estimated glomerular filtration rate.

**Table 3:** Univariate analysis of the associations between clinical characteristics and significant postoperative kidney dysfunction persistence.

Univariate					
Variables	SPKD	Non-SPKD	OR	95% CI	*p*-Value
Age	71.0 (12.0)	63.0 (13.0)	1.07	1.01–1.15	0.022
CCI score	5.0 (2.0)	4.0 (2.0)	1.57	1.06–2.42	0.031
No metabolic syndrome	3 (14.3)	30 (42.9)	0.22	0.05–0.73	0.024
Preoperative eGFR	72.0 (22.0)	90.0 (12.5)	0.91	0.86–0.95	<0.001
Preoperative uACR	2.5 (0.9)	1.3 (1.6)	2.18	1.24–4.07	0.009
Warm ischemia time	19.0 (6.0)	14.0 (8.0)	1.14	1.05–1.27	0.005
Estimated blood loss > 500 mL	10 (47.6)	6 (8.6)	9.70	3.02–34.07	<0.001
Intraoperative hypotension time	40.0 (15.0)	0.0 (13.8)	1.09	1.05–1.14	<0.001
Removed parenchymal volume	34.7 (16.8)	19.0 (15.3)	1.06	1.03–1.11	0.001
Resected part volume	87.1 (53.3)	37.9 (54.8)	1.01	1.00–1.02	0.020
NLR ≤ 3.5	2 (9.5)	36 (51.4)	0.08	0.01–0.33	<0.002
NLR > 9	2 (9.5)	11 (15.7)	0.03	0.01–0.21	0.02

Abbreviations: SPKD—significant postoperative kidney dysfunction; OR—odds ratio; CI—confidence interval; CCI—Charlson comorbidity index; eGFR—estimated glomerular filtration rate; eGFR—estimated glomerular filtration rate; uACR—urine albumin-creatinine ratio; NLR—neutrophil-to-lymphocyte ratio.

**Table 4:** Factors predicting clinically significant postoperative acute kidney injury.

Multivariate			
Variables	OR	95% CI	*p*-Value
Preoperative eGFR ≥ 90 mL/min	0.31	0.10–0.85	0.03
Intraoperative hypotension	1.06	1.03–1.09	<0.001
Parenchymal loss < 20 cm^3^	0.22	0.06–0.71	0.01
Metabolic syndrome			
Ischemia time			
Estimated blood loss			
NLR after 48 h			
Tumor volume			

The OR and 95% CI were measured through ordered logistic regression. Likelihood ratio test statistic is 42.86 (distributed chi-squared), *p* < 0.0001; Pseudo-R² (McFadden) = 0.25; AIC = 132.39; AIC = 175.93 (for model with constant only (no regressors). Abbreviations: OR—odds ratio; CI—confidence interval; eGFR—estimated glomerular filtration rate; NLR—neutrophil to lymphocyte ratio; AIC—Akaike information criteria.

## Data Availability

The datasets generated during the current study are available from the corresponding author upon reasonable request.

## Supplementary Materials

The following supporting information can be downloaded at: https://www.mdpi.com/article/10.3390/medicina58050667/s1, Table S1: Study patients perioperative, intraoperative and postoperative laboratory data; Figure S1: The graphics of postoperative renal function on eGFR.
