# Supplementary material for: Evaluation of Renal Function after Partial Nephrectomy and Detection of Clinically Significant Acute Kidney Injury
Source: Medicina (Kaunas). 2022 May 17;58(5):667. doi: 10.3390/medicina58050667 (PMC9144406; doi:10.3390/medicina58050667)
Supplement: Supplementary file 1 [file medicina-58-00667-s001.zip › Figure S1.medicina-1646128-supplementary.pdf]

1. The effect of postoperative AKI on eGFR

A. After 6 months

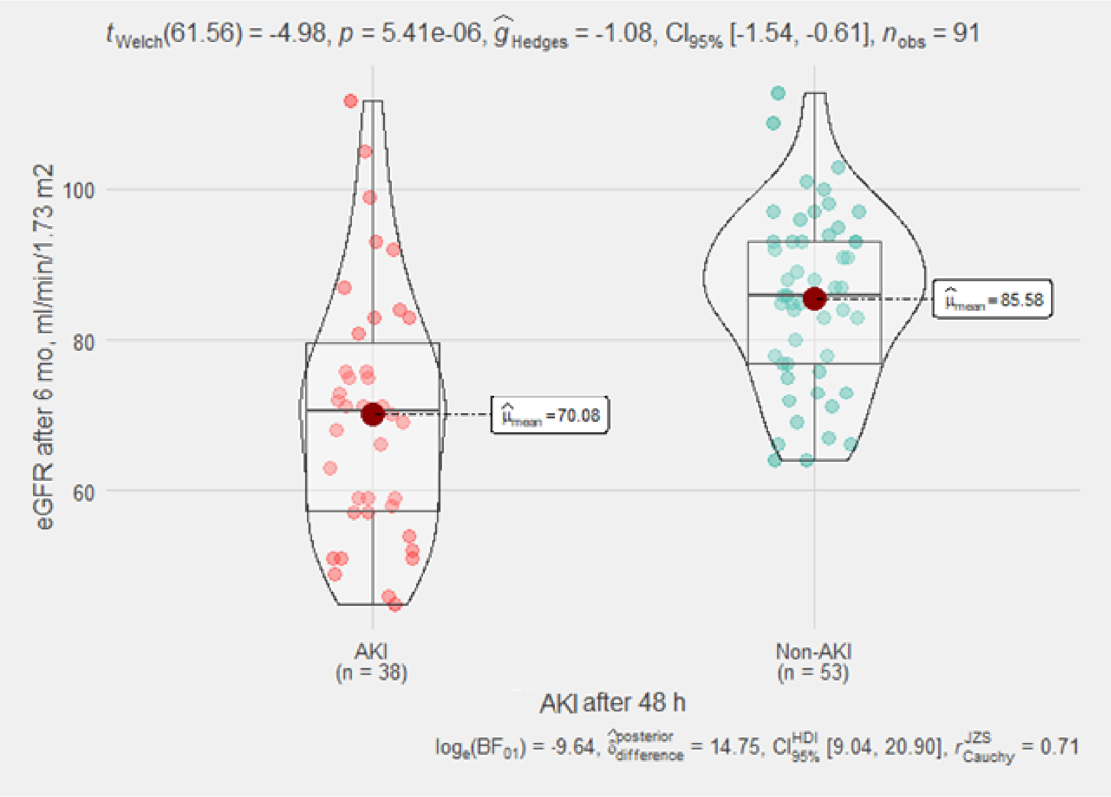

B. After 12 months

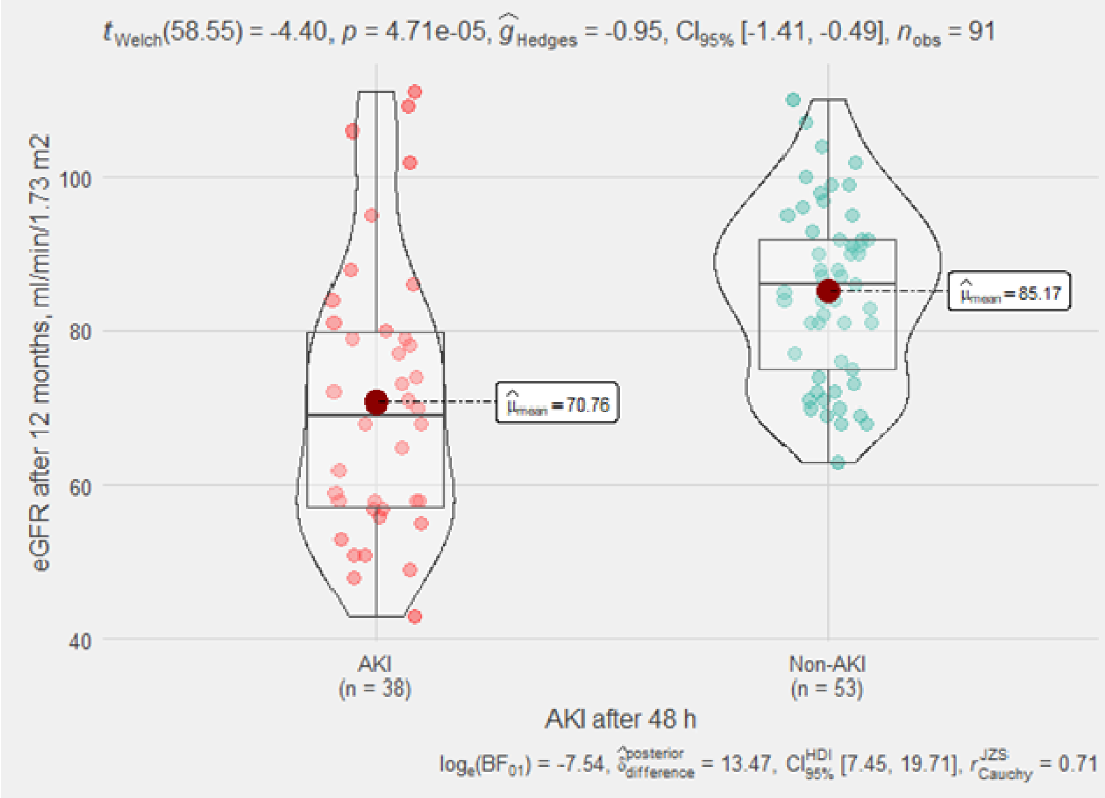

2. The effect of postoperative renal dysfunction on eGFR

C. After 6 months

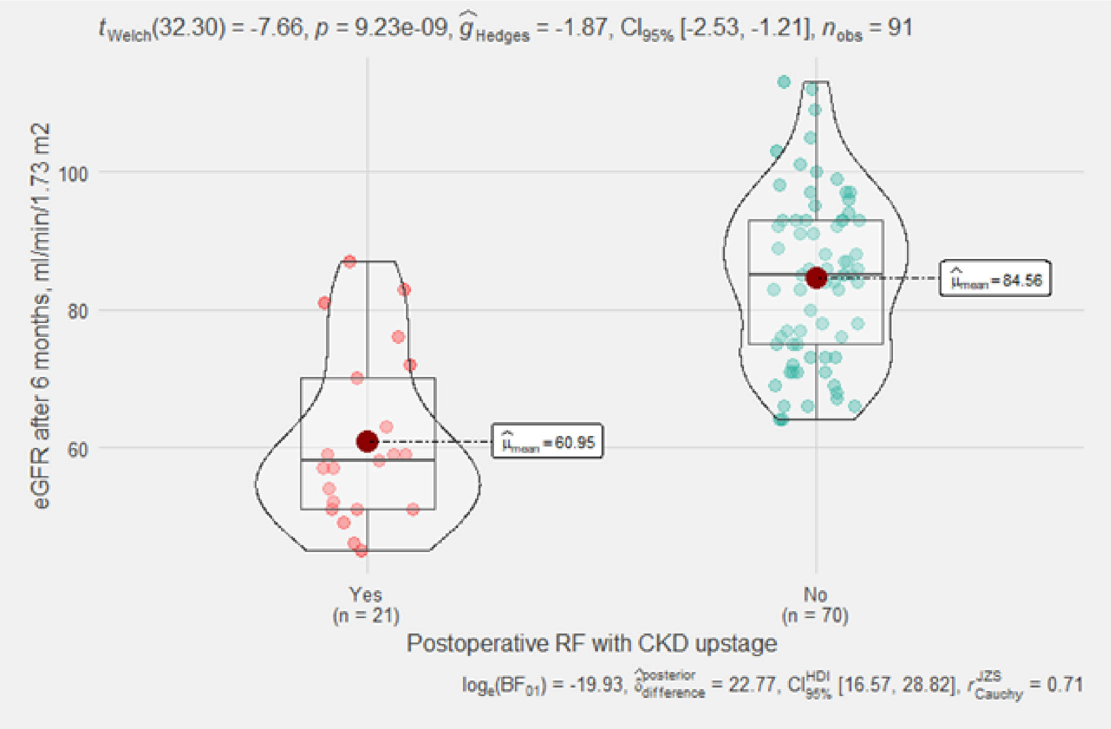

D. After 12 months

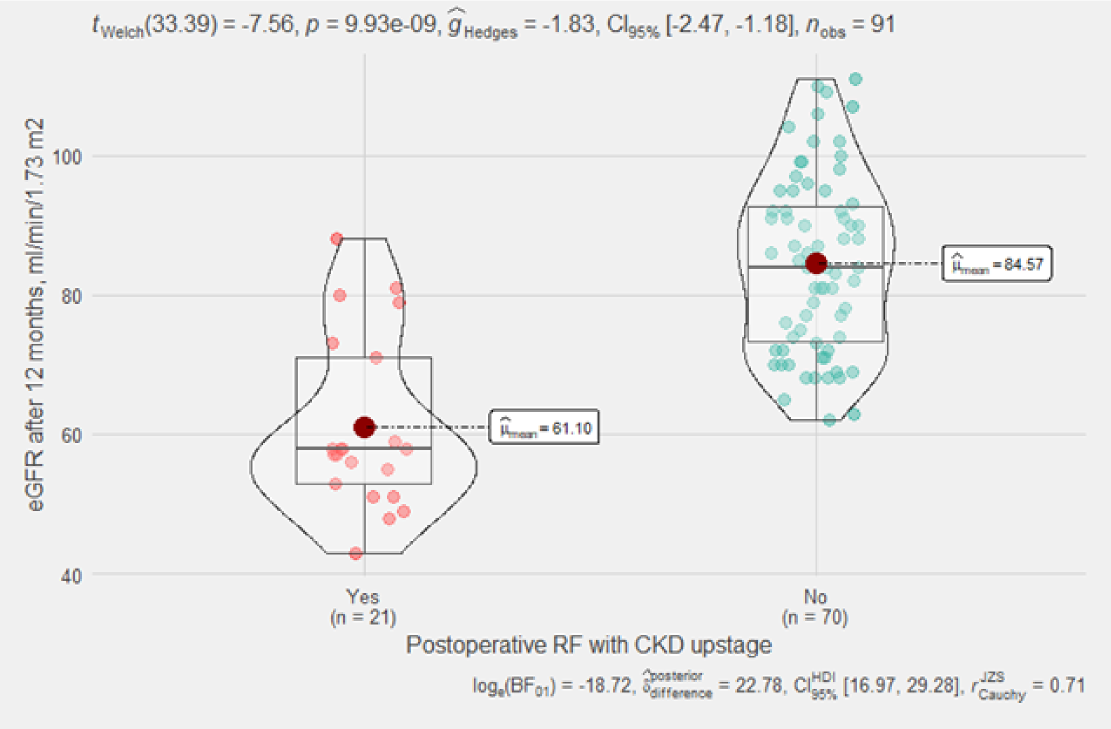

3. The effect of postoperative AKI status on eGFR

E. After 6 months

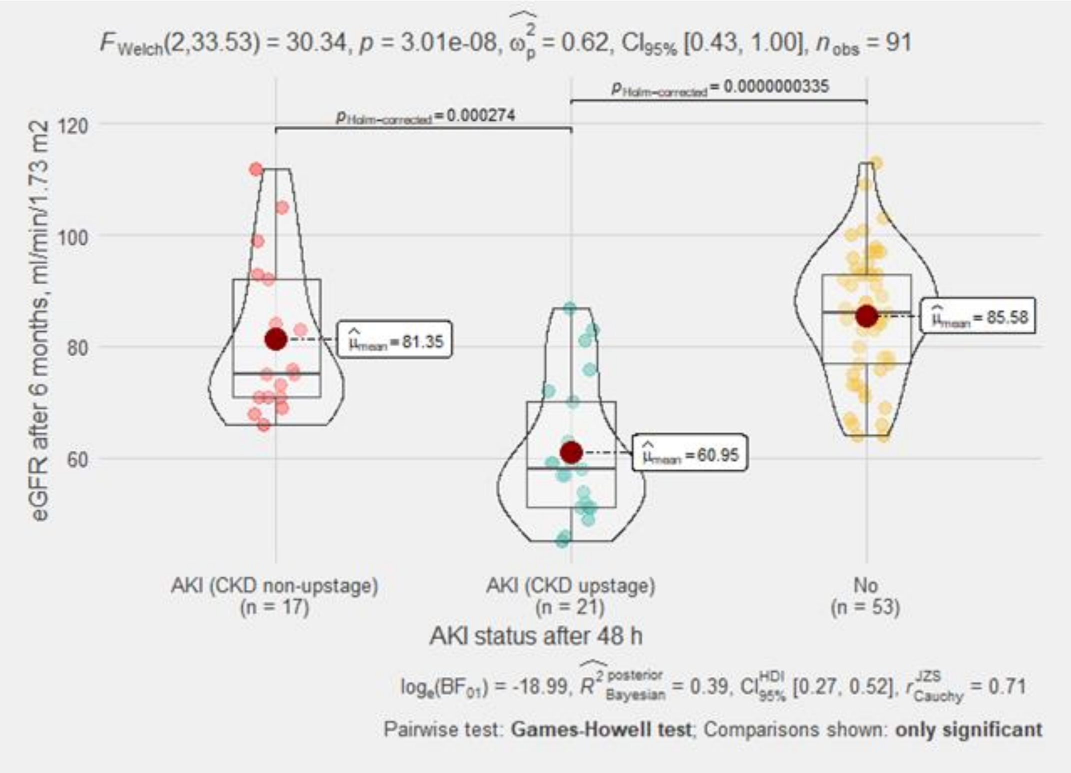

E. After 12 months

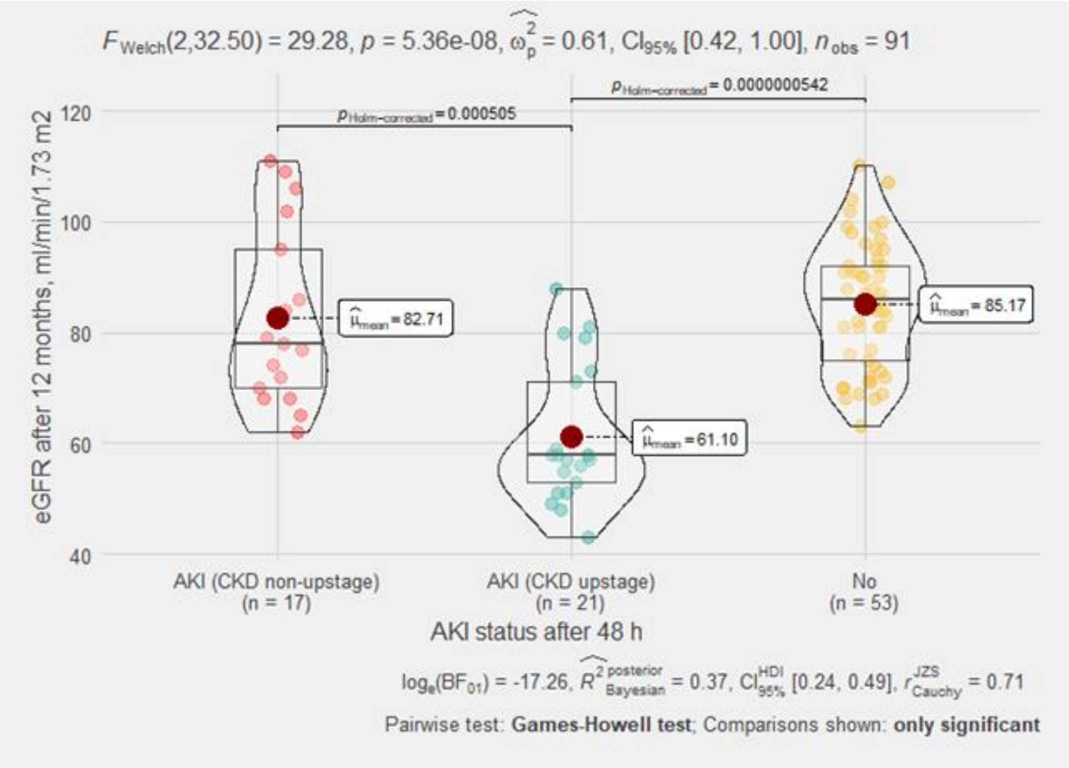

Figure S1. The graphics of postoperative renal function on eGFR.

Welch's t-test revealed that, across 91 patients, although the eGFR after 6 months, ml/min/1.73 m^2: **A** - were lower in AKI group after 48 h, as compared to non-AKI group. This effect was statistically significant. The effect size (g=-1.08)(p<0.0001) was hight, as per Cohen's (1988) conventions. The Bayes Factor for the same analysis revealed that the data were 15 times more probable that the means are different as compared to the null hypothesis, that the means are equal; **C** - were lower in postoperative renal dysfunkcion group, as compared to non-dysfunction group. This effect was statistically significant. The effect size (g=-1.87)(p<0.0001) was hight, as per Cohen's (1988) conventions. The Bayes Factor is 23; **E** - were lower in AKI with CKD upstage group after 48 h, as compared to non-AKI and AKI without CKD upstage groups. This effect was statistically significant. The effect size (g=-0.62)(p<0.0001) was hight. Welch's t-test revealed that, across 91 patients, although the eGFR after 12 months, ml/min/1.73 m^2: **B** - were lower in AKI group after 48 h, as compared to non-AKI group. This effect was statistically significant. The effect size (g=-0.95)(p<0.0001) was hight.The Bayes Factor is 13; **D** - were lower in postoperative renal dysfunkcion group, as compared to non-dysfunction group. This effect was statistically significant. The effect size (g=-1.83)(p<0.0001) was hight, as per Cohen's (1988) conventions. The Bayes Factor is 23; **F** - were lower in AKI with CKD upstage group after 48 h, as compared to non-AKI and AKI without CKD upstage groups. This effect was statistically significant. The effect size (g=-0.62)(p<0.0001) was hight.
