# Supplementary material for: Evaluation of Renal Function after Partial Nephrectomy and Detection of Clinically Significant Acute Kidney Injury
Source: Medicina (Kaunas). 2022 May 17;58(5):667. doi: 10.3390/medicina58050667 (PMC9144406; doi:10.3390/medicina58050667)
Supplement: Supplementary file 1 [file medicina-58-00667-s001.zip › Table S1.medicina-1646128-supplementary.pdf]

Table S1. Study patients perioperative, intraoperative and postoperative laboratory data.

Variable	Value	AKI (CKD upstage)	AKI (CKD non-upstage)	Non-AKI	p - value (Fisher's)
Preoperative eGFR ml/min/1.73 m^2	Median (IQR)	72.0 (22.0)	87.0 (12.8)	93.0 (11.0)	<0.001
Preoperative eGFR categories	Normal or high	6 (28.6)	5 (27.8)	31 (59.6)	0.014
	Mildly decreased	15 (71.4)	13 (72.2)	21 (40.4)	
Preoperative uACR mg/mmol	Median (IQR)	2.5 (0.9)	0.8 (2.2)	1.3 (1.2)	0.038
NLR after 48 h	Median (IQR)	4.5 (2.7)	9.6 (6.9)	3.3 (1.3)	<0.001
NLR after 48 h, categories	Normal	2 (9.5)	2 (11.1)	34 (65.4)	<0.001
	Increased	17 (81.0)	8 (44.4)	18 (34.6)	
	Severely increased	2 (9.5)	8 (44.4)		
sCr after 48.h, μmol/l	Median (IQR)	107.0 (22.0)	106.5 (33.0)	79.0 (14.0)	<0.001
eGFR after 48 h, ml/min/1.73 m^2	Median (IQR)	51.0 (15.0)	56.0 (22.0)	83.0 (20.0)	<0.001
eGFR after 48 h, categories	Normal or high			22 (42.3)	<0.001
	Mildly decreased	5 (23.8)	7 (38.9)	28 (53.8)	
	Mildly to moderately decreased	10 (47.6)	9 (50.0)	2 (3.8)	
	Moderately to severely decreased	5 (23.8)	2 (11.1)		
	Severely decreased	1 (4.8)			
NLR after 2 months	Median (IQR)	1.8 (0.5)	1.8 (1.2)	1.7 (0.6)	0.672
sCr after 2 months, μmol/l	Median (IQR)	95.0 (23.0)	89.0 (23.5)	77.0 (13.5)	<0.001
eGFR after 2 months, ml/min/1.73 m^2	Median (IQR)	57.0 (16.0)	76.5 (12.5)	87.0 (14.8)	<0.001
eGFR after 2 months, categories	Mildly decreased	8 (38.1)	15 (83.3)	30 (57.7)	<0.001

	Normal or high		3 (16.7)	22 (42.3)	
	Mildly to moderately decreased	12 (57.1)			
	Moderately to severely decreased	1 (4.8)			
sCr after 6 months, μmol/l	Median (IQR)	96.0 (20.0)	83.5 (16.5)	75.5 (16.0)	<0.001
eGFR after 6 months, ml/min/1.73 m2	Median (IQR)	58.0 (19.0)	75.5 (19.0)	86.0 (16.0)	<0.001
eGFR categories after 6 months	Normal or high		5 (27.8)	20 (38.5)	<0.001
	Mildly decreased	7 (33.3)	13 (72.2)	32 (61.5)	
	Mildly to moderately decreased	14 (66.7)			
sCr after 12 months, μmol/l	Median (IQR)	97.0 (22.0)	79.0 (15.2)	74.0 (20.0)	<0.001
eGFR after 12 months, ml/min	Median (IQR)	58.0 (18.0)	78.5 (22.2)	86.5 (17.5)	<0.001
eGFR categories after 12 months	Mildly decreased	6 (28.6)	13 (72.2)	30 (57.7)	<0.001
	Normal or high		5 (27.8)	22 (42.3)	
	Mildly to moderately decreased	14 (66.7)			
	Moderately to severely decreased	1 (4.8)			
uACR after 12 months, mg/mmol	Median (IQR)	4.9 (3.7)	2.0 (3.4)	1.8 (1.8)	0.007

Abbreviations: IQR – interquartile range; eGFR – estimated glomerular filtration rate; uACR – urine albumin-creatinine ratio; NLR - neutrophil to lymphocyte ratio; sCr – serum creatinine. *p*-values calculated for comparison of non-AKI, AKI without CKD upstage and AKI with CKD upstage cohorts.
